# The role of dietary fat in prostate cancer risk in Jamaican men: a pilot study

**DOI:** 10.1186/1750-9378-6-S1-A5

**Published:** 2011-08-11

**Authors:** Ayokunle Osho, Tirsit Adane, Maung Aung, Flora AM Ukoli, Derrick J Beech

**Affiliations:** 1 Department of Surgery, Meharry Medical College, Nashville, Tennessee, USA; 2 Western Regional Health Authority, Montego Bay, Jamaica, WI

## Background

African-American and Jamaican men record the highest prostate cancer rates in the world. Both genetic and environmental factors have been implicated in this increased risk profile. This pilot study was conducted to evaluate the feasibility of a case-control study in Jamaica, describe the demographic characteristics, prostate symptom profile, and dietary fat consumption pattern of men attending the clinics of Cornwall Regional Hospital, Montego Bay, Jamaica.

## Methods

Over a 3-week period men attending the clinics were approached to volunteer to complete a demographic, urology symptom and dietary assessment surveys by interview. The first 40 men to sign informed consent were recruited into the study.

## Results

This study involved men between the ages of 24 to 88 with an average age of 52years. Twenty percent of the men had a college degree, 47.5% had a high school diploma. Half of the subjects were married, 40% were employed fulltime 30% were obese. Seven of the 40 subjects (17.5%) and 22.5% reported a previous diagnosis of prostate cancer and enlarged prostate respectively.

## Conclusion

A case-control study to investigate the role of dietary fat in prostate cancer risk among Jamaican men is feasible in this hospital. Dietary fat consumption patterns are different across education and age groups, providing the opportunity to evaluate its impact on prostate cancer risk. A modified BLOCK FFQ that includes ethnic Jamaican food items will be required to capture the eating pattern in this population in more detail.

